# Hypoglycemia associated with L-asparaginase in acute lymphoblastic leukemia treatment: a case report

**DOI:** 10.1186/2162-3619-1-8

**Published:** 2012-04-19

**Authors:** Ryuma Tanaka, Tomoo Osumi, Masashi Miharu, Tomohiro Ishii, Tomonobu Hasegawa, Takao Takahashi, Hiroyuki Shimada

**Affiliations:** 1 Department of Pediatrics, Keio University School of Medicine, 35 Shinanomachi, Shinjuku-ku, Tokyo, 160-8582, Japan

**Keywords:** L-asparaginase, Fasting hypoglycemia, Hyperinsulinism, Acute lymphoblastic leukemia, Fasting Glucose levels

## Abstract

A patient with acute lymphoblastic leukemia repeatedly developed hypoglycemia during chemotherapy. Comparison of serum glucose trends between chemotherapy with and without L-asparaginase (L-Asp) demonstrated a strong association between L-Asp and hypoglycemia. Critical blood sampling during hypoglycemia indicated hyperinsulinism, suggesting that L-Asp induced hypoglycemia in the patient through inappropriate insulin secretion. Identification of hypoglycemia as an adverse effect will enable clinicians to understand and develop appropriate strategies for L-Asp use in chemotherapy regimens.

## Background

The enzyme L-asparaginase (L-Asp) has been commonly used for treatment of childhood acute lymphoblastic leukemia (ALL) for more than 30 years [1-3]. Because of its unique pharmacological features and historically improved treatment outcomes, L-Asp forms an essential part of ALL regimens worldwide [4]. However, many adverse effects of L-Asp have been documented, such as coagulopathy, acute pancreatitis, allergic reaction, hyperlipidemia, hyperammonemia, hepatotoxicity, and hyperglycemia [5-8]. Therefore, clinicians must carefully monitor patients treated with L-Asp for these adverse effects. Until date, hypoglycemia has not been formally reported as an adverse effect of L-Asp. We report, for the first time, a case of hypoglycemia associated with L-Asp use.

## Case presentation

**Figure 1:**
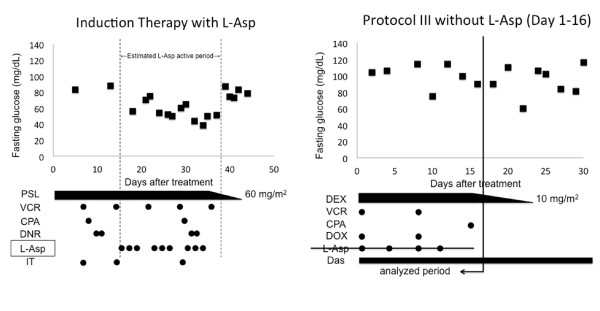
**Fasting serum glucose levels during induction therapy with L-Asp and protocol III without L-Asp (Day 1–16).** Monitoring of fasting serum glucose shows hypoglycemia during the estimated L-asp active period, displayed as the zone between dashed lines (——−−−−). PSL denotes predonisolone; VCR, vincristine; CPA, cyclophosphamide; DNR, daunorubicin; L-Asp, L-asparaginase; IT, intrathecal injection; DEX, dexamethasone; DOX, doxorubicin; and Das, dasatinib.

A 5-year-old girl with Philadelphia chromosome-positive ALL was treated with the induction therapy protocol of the Tokyo Children’s Cancer Study Group (TCCSG) L99-15 for high-risk patients. Therapy included prednisolone, vincristine, cyclophosphamide, daunorubicin, triple intrathecal injection, and L-Asp (Kyowa Hakko Kirin, Tokyo, Japan) [9]. The patient was administered 6000 IU/m^2^ of native *Escherichia coli* L-Asp on days 16, 18, 20, 23, 25, 27, 30, 32, and 34 of treatment. On day 18, she developed fasting hypoglycemia (glucose, 56 mg/dL) with severe hunger and without other signs or symptoms such as tremor, palpitation, anxiety, sweating, or paresthesias. On day 27 (fasting serum glucose level, 50 mg/dL), critical blood sampling showed 6 U/mL of immunoreactive insulin, 0.47 mEq/L of free fatty acids, and 27 mol/L of ketone bodies. She repeatedly developed fasting hypoglycemia (glucose, 38–65 mg/dL) until day 37, without serious complications (Figure 1). Five days after the last L-Asp administration, her fasting glucose levels were elevated from 73 to 87 mg/dL. During induction therapy, fasting glucose levels were always measured before the morning steroid dose.

TCCSG L99-15 intensification-1, including high-dose cytarabine, methylprednisolone, L-Asp, and triple intrathecal injection, was started with daily imatinib. Thereafter, HR blocks of Berlin–Frankfurt–München (BFM) 2000 were administered. The HR blocks included cytarabine, etoposide, methotrexate, vindesine, ifosfamide, daunorubicin, cyclophosphamide, dexamethasone, and L-Asp [10]. Daily dasatinib was included in the HR blocks instead of imatinib to avoid allergic reaction during TCCSG intensification-1. L-Asp was administered on day 5 of intensification-1 and days 6 and 11 of the HR block therapy. During early treatment days before L-Asp administration, the patient repeatedly developed fasting hyperglycemia (glucose, up to 132 mg/dL). After L-Asp administration, she repeatedly experienced fasting hypoglycemia and was provided with supplemental food and intravenous fluid containing dextrose to prevent severe hypoglycemia.

At the end of the third HR block, she suffered acute pancreatitis. When chemotherapy was resumed after 2 weeks of treatment for pancreatitis, L-Asp was excluded from protocol III of BFM 2000 (reinduction therapy), which included dexamethasone, vincristine, doxorubicin, cyclophosphamide, cytarabine, mercaptopurine, and intrathecal methotrexate administration. During protocol III, the patient did not experience hypoglycemia and had relatively high fasting glucose levels, up to 114 mg/dL (Figure 1).

**Figure 2:**
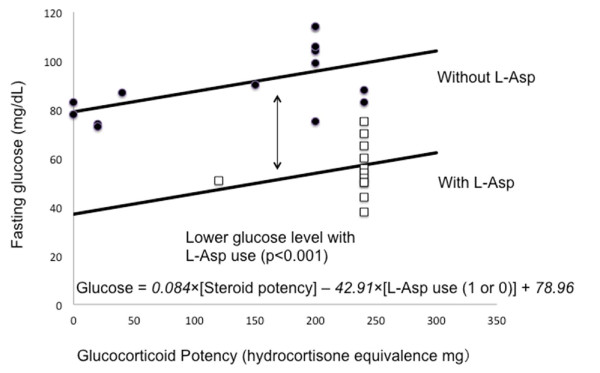
**Linear regression analysis of fasting glucose, L-Asp, and glucocorticoid potency during induction therapy and protocol III.** Linear regression analysis shows significant hypoglycemia during L-Asp use (*p* < 0.001) and a significant hyperglycemic effect of glucocorticoids (*p* = 0.015). Fasting glucose levels were plotted with L-Asp (□) and without L-Asp (●). Glucocorticoid potency is calculated as hydrocortisone equivalence with conversion ratios of 4:1 (prednisolone) and 20:1 (dexamethasone). L-Asp denotes L-asparaginase.

Glucose levels during the estimated L-Asp active period were significantly lower than those before and after the induction therapy period (Student *t*-test, p < 0.05). The estimated L-Asp active period was defined as that from the first administration to 4 days after the last administration during induction therapy, given that the patient’s fibrinogen level decreased with L-Asp and spontaneously recovered 5 days after the last L-Asp administration. This estimation is supported by a report that L-Asp activity is retained for 3 days in the BFM 2000 protocol, where 5000 IU/mL L-Asp is administered at 3-day intervals [11]. Thereafter, a linear regression model was used to identify the relationship between fasting glucose levels, L-Asp, and glucocorticoid potency during induction therapy and protocol III, with the same treatment structure as that of induction therapy. Variables were entered into a least-squares model. Prednisolone and dexamethasone potency was converted to hydrocortisone potency using 4:1 and 20:1 ratios, respectively [12]. Fasting glucose levels before and after the estimated L-Asp active period of induction therapy and during days 1–16 of protocol III were treated as values without L-Asp, whereas fasting glucose levels during the estimated L-Asp active period were treated as values with L-Asp. Linear regression analysis showed that fasting glucose levels were significantly lower during the L-Asp active period (42.91 mg/dL, p < 0.001), and the hyperglycemic effect of glucocorticoids was significant (glucose, 0.084 mg/dL per mg hydrocortisone equivalence, p = 0.015), indicating the following equation: glucose = 0.084 × [steroid potency] − 42.91 × [L-Asp use (1 or 0)] + 78.96 (Figure 2).

We confirmed that L-Asp activity was therapeutic during fasting hypoglycemia in the first HR block. L-Asp activity was 0.709 U/mL on day 10, 0.674 U/mL on day 11, and 1.78 U/mL on day 12, where 25000 U/m^2^ of L-Asp was administered on days 6 and 11. L-Asp activity was measured using the enzyme coupling methods described by Tsurusawa et al. [13,14]. L-Asp activity above 0.1 U/mL was generally considered therapeutic. The detection limit of this method was 0.002 U/mL.

## Conclusions

During ALL treatment, glucose levels are routinely monitored because many patients develop hyperglycemia, presumably because of glucocorticoids and L-Asp. Unexpectedly, our patient experienced repeated fasting hypoglycemia during induction therapy, intensification-1, and HR block therapy. From induction through HR block therapy, L-Asp and steroids were both used and hypoglycemia was always observed after L-Asp use. Given the time relation between L-Asp administration and hypoglycemia, we hypothesized that our patient experienced L-Asp–induced hypoglycemia. Firstly, we compared fasting glucose levels with L-Asp and without L-Asp during induction (Student *T*-test), showing that those with L-Asp were significantly lower. After HR block therapy, L-Asp was excluded from protocol III because of the development of pancreatitis. Then, we compared fasting glucose levels during induction and first half of protocol III, the same backbone of treatment with and without L-Asp, respectively.

The following questions are to be answered: 1) How does L-Asp induce hypoglycemia? 2) Is there any use of other agents that might affect fasting glucose levels? 3) Why has hypoglycemia not been commonly seen or reported?

In response to the first question, inappropriate insulin secretion, normal free fatty acids, and low ketone bodies during severe hypoglycemia indicated hyperinsulinism, suggesting that L-Asp induced hypoglycemia through insulin hypersecretion. Regarding the second question, besides L-Asp use, there were the following differences between induction therapy and day 1–16 of protocol III; doxorubicin was used in protocol III instead of daunorubicin in induction therapy, and dasatinib was used in protocol III but not in induction therapy. Dasatinib has been not documented to cause the adverse effect of hyperglycemia or hypoglycemia. There was no use of other drugs that potentially induce hypoglycemia or hyperglycemia during these periods, such as non-steroidal anti-inflammatory drugs, antibacterial agents, or imatinib [15]. To the third question, there are few reports focusing on fasting glucose levels in the morning [6,7]. Glucocorticoids are known to induce hyperglycemia through insulin resistance and gluconeogenesis during induction therapy for childhood ALL [16]. We suspect that parallel use of glucocorticoids may mask the hypoglycemic effect of L-Asp. Since different glucocorticoid agents were used in these two regimens, we treated glucocorticoids as an independent parameter with glucocorticoid potency conversion. Linear regression model showed a significant L-Asp hypoglycemic effect as well as glucocorticoid hyperglycemic effect. There was no significant interaction between L-Asp and glucocorticoids. There remains a concern with validity of glucocorticoid potency conversion for hyperglycemic effect. To overcome the limitation of this case report, further clinical data focusing on hypoglycemia are needed.

Use of L-Asp has recently become even more common in the adult ALL regimens [17]. We believe that in addition to the effect of L-Asp on leukemic cells, it is important to pay more attention to its pharmacological features and physiological mechanisms. Greater knowledge of these effects will enable clinicians to understand and develop appropriate strategies for L-Asp use in chemotherapy regimens.

### Consent

Written informed consent was obtained from the patient’s guardian for publication of this case report. A copy of the written consent is available for review by the Editor-in-Chief of this journal.

## Abbreviations

L-Asp, L-asparaginase; ALL, Acute lymphoblastic leukemia; TCCSG, Tokyo Children’s Cancer Study Group; BFM, Berlin–Frankfurt–München.

## Competing interests

The authors declare that they have no competing interests.

## Authors’ contributions

RT designed and wrote the paper. TO, MM, and HS were responsible for the patient’s overall treatment and review of the manuscript. TI and TH were responsible for the patient’s endocrinological management and investigations. TT and HS performed their critical interpretation. All authors read and approved the final report.

## Authors’ information

Department of Pediatrics, Keio University School of Medicine, Tokyo, Japan
